# 

**Published:** 1910-08

**Authors:** 


### Contributors to Vol. LXVI

**AUGUST, 1910—JULY, 1911**

---

| | |
|---|---|
| Benedict, A. L. | Buffalo |
| Bethune, Charles W. | Buffalo |
| Coleman, N. R. | Columbus |
| Connor, Leartus | Detroit |
| Fronczak, F. E. | Buffalo |
| Frost, W. H. | Washington |
| Gaylord, H. R. | Buffalo |
| Gould, G. M. | Ithaca |
| Gram, F. C. | Buffalo |
| Greenleaf, C. R. | Hornell |
| Haase, C. | Elmira |
| Hanley, L. G. | Buffalo |
| Hayd, H. E. | Buffalo |
| Hopkins, H. R. | Buffalo |
| Kauffman, L. | Buffalo |
| Long, Eli H. | Buffalo |
| Lovett, R. W. | Boston |
| MacLeod, J. A. | Buffalo |
| MacLeod, N. K. | Buffalo |
| McGuire, Edgar R. | Buffalo |
| McMurtry, L. S. | Louisville |
| Mann, M. D. | Buffalo |
| Matson, G. H. | Columbus |
| Mason, F. S. | New York |
| Mills, W. W. | West New Brighton |
| Park, Roswell | Buffalo |
| Pryor, J. H. | Buffalo |

| | |
|---|---|
| Putnam, James W. | Buffalo |
| Quirk, J. M. | Montour Falls |
| Rafter, J. A. | Buffalo |
| Schroeter, L. | Buffalo |
| Sears, F. W. | Syracuse |
| Stockton, Charles G. | Buffalo |
| Tinker, M. B. | Ithaca |
| VanPeyma, P. W. | Buffalo |
| VanBlarcon, C. C. | New York |
| Wallace, W. L. | Syracuse |
| Webster, J. C. | Chicago |
| Wende, G. W. | Buffalo |
| Wilson, N. W. | Buffalo |
| Woehnert, A. E. | Buffalo |
| Wright, T. | Buffalo |
| Young, A. A. | Newark, N. Y. |

American Medical Association, Conference on Medical Education

Association of American Medical Colleges

Buffalo Academy of Medicine

Medical Association of Central New York

Medical Society County of Chemung

Medical Society County of Erie

Medical Society County of Schuyler

National Confederation State Medical Examining and Licensing Boards

### BOOKS RECEIVED

American Practice of Surgery. A complete System of the Science and Art of Surgery by representative surgeons of the United States and Canada. Edited by Joseph D. Bryant, M.D., and Albert H. Buck, M.D., New York. Complete in eight volumes. Vol. VII. Imperial octavo, pp. 961. Profusely illustrated. New York: William Wood & Company. 1909. (Price, cloth, $7.00.)

A Manual of Operative Surgery. By Sir Frederick Treves, Bart., G.C.V.O., C.B., LL.D., F.R.C.S., Sergeant-Surgeon to H.M. the King, Surgeon-in-Ordinary to H. R. H. the Prince of Wales, Consulting Surgeon to the London Hospital; and Jonathan Hutchinson, F.R.C.S., Surgeon to the London Hospital. New (3d) Edition, revised and rewritten. In two octavo volumes. Volume II, 820 pages, with 302 engravings, and 8 full-page plates. Lea & Febiger, Publishers, Philadelphia and New York, 1910. (Half-morocco, $6.50, net.)

The Practical Medicine Series. Ten volumes. Under the general editorial charge of Gustavus P. Head, M.D., Professor of Laryngology and Rhinology in the Chicago Post-Graduate Medical School. Vol. I, General Medicine, edited by Frank Billings, M.D., and J. H. Salisbury, M.D. Vol. II, Eye, Ear, Nose and Throat, edited by Casey A. Wood, M.D., Albert H. Andrews, M.D., and G. P. Head, M.D. Vol. III, General Surgery, edited by John B. Murphy, M.D. Series 1910. Chicago: The Year Book Publishers. (Prices, $1.50, $1.50 and $2.00; entire series, $10.00.

Nursing in Diseases of the Eye, Ear, Nose, and Throat. By the Committee on Nurses of the Manhattan Eye, Ear, and Throat Hospital, New York City. 12mo volume of 281 pages, illustrated. Philadelphia and London: W. B. Saunders Company, 1910. (Cloth, $1.50 net.)

Dyspepsia: Its Varieties and Treatment. By W. Soltau Fenwick, M.D. (London), Doctor of Medicine of the University of Strassburg. Octavo of 485 pages, illustrated. Philadelphia and London: W. B. Saunders Company, 1910. (Cloth, $3.00 net.)

International Clinics. A Quarterly of Illustrated Clinical Lectures and especially prepared articles on Treatment, Medicine, Surgery, Neurology, Pediatrics, Obstetrics, Gynecology, Orthopedics, Pathology, Dermatology, Ophthalmology, Otology, Rhinology, Laryngology, Hygiene, etc. Edited by Henry W. Cattell, M.D. Vol II, twentieth series. Philadelphia and London: J. B. Lippincott Co. 1910. (Cloth, $2.00.)

Transactions of the American Dermatological Association at its thirty-third annual meeting held in Philadelphia, June 3-5, 1909. Grover W. Wende, secretary.

